# A Precocious Cerebellar Ataxia and Frequent Fever Episodes in a 16-Month-Old Infant Revealing Ataxia-Telangiectasia Syndrome

**DOI:** 10.1155/2013/296827

**Published:** 2013-10-28

**Authors:** Luigi Nespoli, Annapia Verri, Silvia Tajè, Francesco Paolo Pellegrini, Maddalena Marinoni

**Affiliations:** ^1^Pediatrics Unit, Department of Clinical and Experimental Medicine, University of Insubria, 21100 Varese, Italy; ^2^Neurological Institute C. Mondino Foundation IRCCS, 27100 Pavia, Italy

## Abstract

Ataxia-telangiectasia (AT) is the most frequent progressive cerebellar ataxia in infancy and childhood. Immunodeficiency which includes both cellular and humoral arms has variable severity. Since the clinical presentation is extremely variable, a high clinical suspicion will allow an early diagnosis. Serum alpha-fetoprotein is elevated in 80–85% of patients and therefore could be used as a screening tool. Here, we present a case of a 5-year-old female infant who was admitted to our department at the age of 16 months because of gait disorders and febrile episodes that had begun at 5 months after the cessation of breastfeeding. Serum alfa-fetoprotein level was elevated. Other investigations showed leukocytopenia with lymphopenia, reduced IgG_2_ and IgA levels, and low titers of specific postimmunization antibodies against tetanus toxoid and Haemophilus B polysaccharide. Peripheral lymphocytes subsets showed reduction of T cells with a marked predominance of T cells with a memory phenotype and a corresponding reduction of naïve T cells; NK cells were very increased (41%) with normal activity. The characterization of the ATM gene mutations revealed 2 specific mutations (c.5692C > T/c.7630-2A > C) compatible with AT diagnosis. It was concluded that AT syndrome should be considered in children with precocious signs of cerebellar ataxia and recurrent fever episodes.

## 1. Introduction

Ataxia-telangectasia (AT) is a complex multisystem disorder characterized by progressive neurological impairment, variable immunodeficiency, and oculocutaneous telangiectasia [1]. AT is a member of chromosomal breakage syndromes caused by a mutation in the ataxia-telangiectasia mutated (ATM) gene [2, 3]. The immunodeficiency is of variable severity in relation to the specific ATM mutation and is associated with sinopulmonary infections, radiation hypersensitivity, and increased incidence of malignancy [3, 4]. Cells from patients show increased sensitivity to ionizing radiation, defective DNA repair, and frequent chromosomal abnormalities [5, 6].

Gait instability with or without recurrent infection is the earliest symptom and oculocutaneous teleangiectasias will appear later [7]. The complete phenotype occurs over a number of years, usually within the school age [8, 9]. An easy and reliable marker in case of suspected AT is the elevated serum level of alpha-fetoprotein which is present in 80–85% of the affected patients [1]. An early diagnosis is possible today by using the molecular approach which will identify the specific ATM mutations and by measuring the ATM protein levels and the ATM kinase activity [3, 4]. AT is transmitted as an autosomal recessive disorder, and its incidence is about 1 per 40.000 live births in the USA, but the frequency varies considerably from country to country [3, 10].

## 2. Case Report

Our patient is a 5-year-old girl who presented to our outpatient service at the age of 16 months with a history of repeated episodes of fever (lasting 3-4 days) of unknown origin sometimes associated with upper respiratory tract infections and oral candidiasis. The episodes subsided without antibiotics and responded to acetaminophen treatment. The infant was not introduced to daycare and she was cared for at home by her grandmother. She began walking just a few weeks before our visit. Her development was within the normal limits. Her parents are from Albania and are nonconsanguineous. Family history was noninformative. She was the first child of the family; the gestational period was normal as well as the birth parameters. Her previous medical history was positive for an exanthema subitum at the age of 8 months. Clinical examination was within the normal range: lymph nodes and intravelic tonsils were present; neurologic examination at that time revealed minimal hypotonia and a gait instability with slightly enlarged basis.

**Figure 1:**
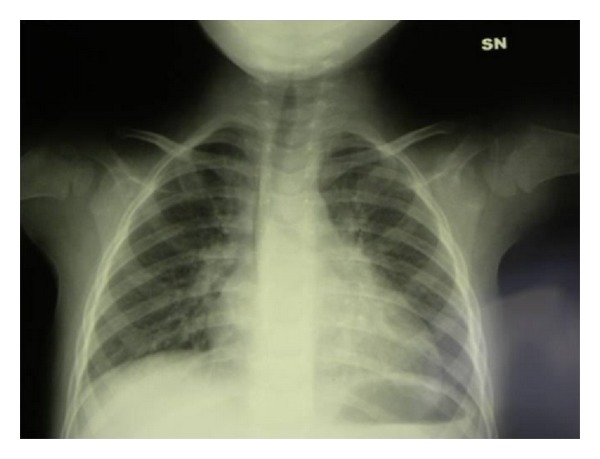
Chest X-ray: the chest X-ray showed reinforcement of the bronchial tree shadows, no bronchiectasis, and a markedly reduced thymic image.

The first blood count showed a leukocyte number at the lower normal limits, microcytic anemia (the mother carries a beta-thalassemia trait), IgG below the lower limits for the age, IgA being absent, and IgM in the normal range. The chest X-ray did show a markedly reduced thymic image (Figure 1).

Our first hypothesis was a combined immunodeficiency; therefore, we performed more in-depth immunological investigations, but in the meantime the patient presented high fever and severe, nonbloody diarrhoea and was hospitalized.

At the admission, her general status was not compromised; she was present, conscious, and reacting to environmental stimuli. Weight was 10.100 kg (50th centile), length was 76.5 cm (50th centile), head circumference was 45 cm (25th centile), hearth rate was 180/min while crying, respiratory rate was 40/min, and ear temperature was 39.2°C; hyperemia was found in the oropharynx and tonsils were visible and covered by grayish exudate with rare rales on the chest; clinical examination of the abdomen and the heart was normal.

Neurological examination revealed ataxic deambulation with frequent falls, forced right deviation, and left rotation of the head with left preferred look and oculomotor apraxia suggestive for cerebellar ataxia. Brain MRI was programmed for the following week and subsequently was normal.

**Figure 2:**
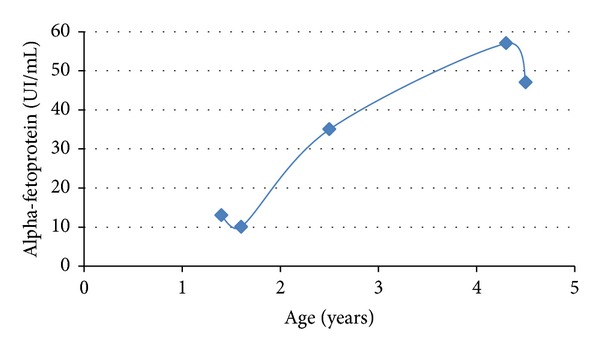
Alpha-fetoprotein trend.

**Figure 3:**
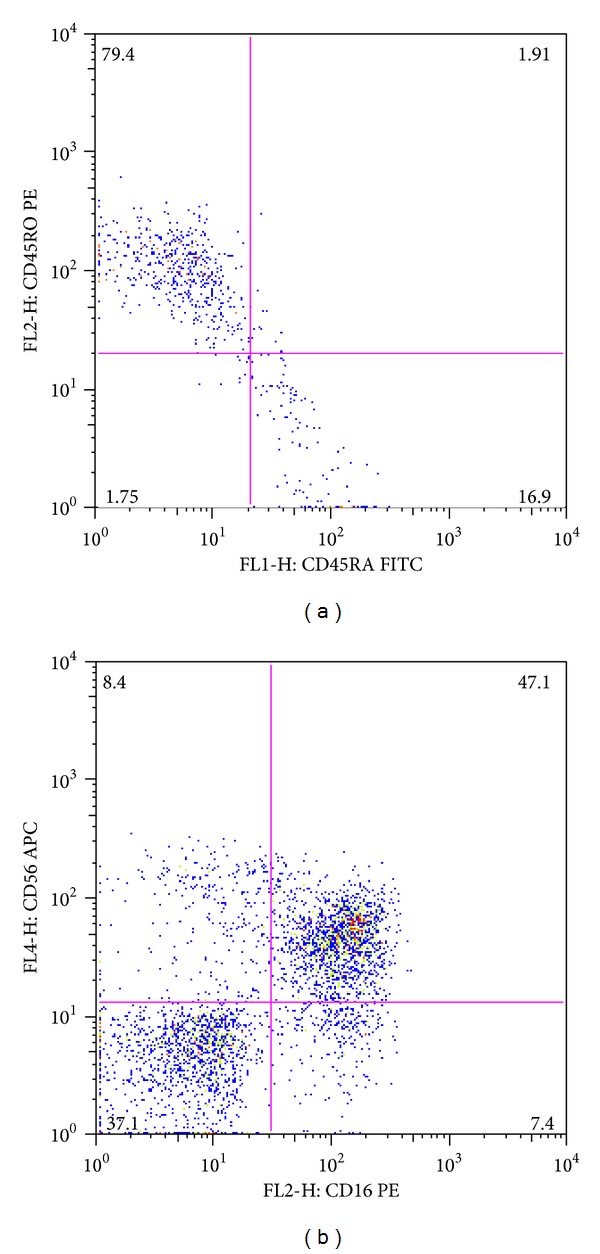
Cytofluorimetric CD45RA/CD45RO ratio (a) and CD16/CD56^+^ population (b). Note the reduced number of naïve T cells (CD45RA) versus peripheral T cells (CD45RO) (17% versus 80%, resp.) and the prevalent NK population (47.1%).

Further laboratory investigations were carried out which showed border line levels of alpha-fetoprotein (13 ng/mL, n.v. < 10 ng/mL). The levels of this protein increased progressively over the years reaching 58 ng/mL when the patient was 4.5 years old (Figure 2). The complete blood count was in the normal range, but after 2 days leucopenia was present (6.820–3.280 WBC/cmm). The absolute number of circulating lymphocytes was below the normal range (Table 1). IgG, IgA, and IgM levels were unchanged as compared to the previous testing (Table 2). Isohemagglutinins, IgM natural antibodies against blood group specific antigens, were absent since the infant carried AB blood group. Postimmunization antibodies against tetanus toxoid and HiB conjugated polysaccharide were present at low levels. Cytofluorimetric analysis showed a low count of CD3^+^ cells and of CD4^+^ and CD8^+^ T cells with a marked predominance of T-memory cells (CD45RO) and a reduction of naïve T cells (CD45RA), an increase of CD16^+^/CD56^+^ cells (NK cells), and a normal number of B cells, CD19^+^ (Table 1 and Figure 3). 

A karyotype test was performed that showed a normal female karyotype with t(7; 14)(p13; q11.2) in 3 metaphases, and t(4; 14)(q13.2; q13) t(5; 6)(p15.3; q23.3) del (7q11.23) in 1 metaphase, respectively.

A blood sample was sent to the laboratory for the DNA molecular study of the ATM gene mutations which revealed 2 specific mutations (c.5692C > T/c.7630-2A > C) compatible with AT diagnosis (mother: c.5692C > T mutation; father: c.7630-2A > C on intron 53).

The child began replacement therapy with i.v. IgG every 21 days to maintain the serum IgG levels within the normal range. This treatment caused a reduction of episodes of fever and infections.

She progressively presented ataxic deambulation with frequent falls, forced right deviation, and left rotation of the head, with left preferred look and motor apraxia. Over a 2-year period, she developed deep gait instability and a difficult feeding due to supervening dysphagia that improved since the 3rd year of life with physiotherapy and exercises. She developed regular relational and cognitive skills and actually she is attending the kindergarten regularly, can eat without problems, walk safely, and drive a tricycle.

**Figure 4:**
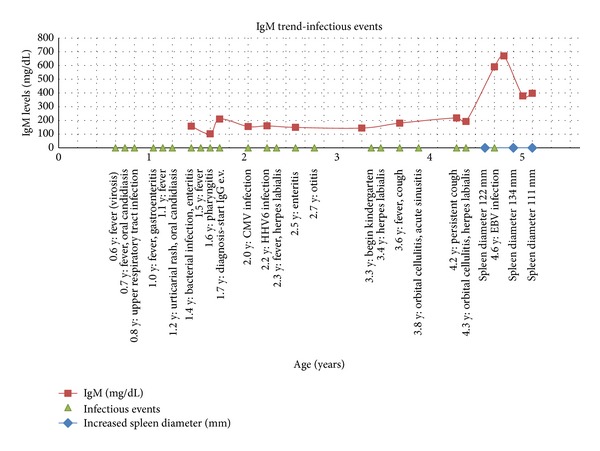
IgM levels according to history infections. Note the increasing levels of IgM simultaneously to EBV-DNA copies and spleen enlargement.

As expected, oculocutaneous telangiectasias became apparent when she was 3 years old. At 4.6 years of age she presented with enlargement of the spleen at the clinical examination which was repeatedly confirmed by the abdominal ultrasound. This fact made us suspect the appearance of a lymphoma. Virological research did document the presence of EBV-DNA (135.000 number of copies/mL) in blood which decreased to 15800 copies/mL after 3 weeks. IgM-specific antibodies were never present in the serum, whereas specific IgG antibodies were detectable but were from the transfused IgG. CMV or other viral DNAs were persistently negative. At the same time, an increase in the IgM serum levels (200→700→370 mg/dL) range was observed (Figure 4) which persisted for several weeks while the spleen size is in reduction but still not within the physiological range.

## 3. Discussion

Patients affected by AT typically present early onset gait disturbances which evolve in typically cerebellar ataxia [1, 7]. The observation of our patient deserves to be taken into account because it shows how careful clinical history together with clinical examination enables us to issue a diagnostic suspicion that today, thanks to molecular studies, can be easily confirmed or denied.

A firm diagnosis has positive effects on the choice of treatment by the doctors and on the procreative planning for the parents [3]. Our patient today has a younger, healthy sister.

Our infant had a history of recurrent fevers seemingly trivial, typical of children of this age, which were associated with an uncertain gait, common in an infant who had just started walking at the age of 15 months. Some peculiarities have led to the diagnosis: febrile episodes were initiated at cessation of breastfeeding at 5-6 months of age which is associated with transient infant hypogammaglobulinemia that had lasted too long in our infant (16-17 months of age when first tested) and was associated with the deficiency of IgA [9]. In addition, history of oral candidiasis is itself an indication of a deficiency of T lymphocytes [11, 12]. The thymic hypoplasia (Figure 1) in association with the low number of circulating lymphocytes further pointed to the diagnosis of combined immunodeficiency [11, 12]. The gait instability suggestive of cerebellar ataxia prompted us to the diagnosis of AT which was further supported by the simultaneous increase in alpha-fetoprotein (13 ng/mL) (Figure 3) [1]. The severity of the immunodeficiency associated with AT is correlated with the reduction of circulating naïve T-cell numbers as observed in our patient [13]. ATM is critically important for the lymphocyte development that relies on double-strand break repair [14, 15] such as V(D)J recombination [16] and class switch recombination of immunoglobulin genes [17, 18].

In our child, as a result of infection with EBV, we observed both an increase in splenic volume and total IgM without, however, the rise of IgM antibodies specific for EBV (Figure 4). Low levels of total IgG and of specific postimmunization IgG antibodies document their defective synthesis in our child. The important reduction of the circulating viral DNA in the child as well as the healing of the previous exanthema subitum, sustained by HHV-6, in our patient demonstrates that the NK cell function is very effective and capable of containing the infections. The increased percentage of circulating NK cells may reflect their increased activity (Figure 3). Even the early onset AT patients referred to in the literature were associated with severe hypogammaglobulinemia and had been diagnosed with isolated severe hypogammaglobulinemia [4, 19]. The precocious introduction of i.v. IgG treatment has allowed our little girl to attend kindergarten without severe infections and to regularly follow the physiotherapeutic treatment. She is now able to speak in an understandable way and to communicate with other children and adults, as well as to walk and ride a tricycle. In fact, to date there is no codified and accepted treatment for neurological manifestations of AT [20], but there are many lines of evidence in other conditions associated with neurological suffering that early therapeutic intervention is able to create new synaptic network in the brain [21].

## 4. Conclusion

AT syndrome should be considered in children with precocious signs of cerebellar ataxia especially if associated with the evidence of defective immune function. A reflection on our case allows us to state that although the diagnostic hypothesis should always start from the clinical observation and the patient history, the possibility of a genetic investigation that is offered to us today is essential for therapeutic intervention to be ready, focused, and motivated.

## Figures and Tables

**Table 1:** Lymphocyte subsets. A low count of CD3^+^ cells and of CD4^+^ and CD8^+^ T cells with an increase of CD16/CD56^+^ cells (NK cells) and a normal number of B cells (CD19^+^) was found in our patient.

Lymphocyte subsets	Absolute number (×10^9^/L)	Reference values (5th–95th centiles)
Total lymphocyte	1776	2180–8270
CD3^+^	578	1460–5440
CD4^+^	340	1020–3060
CD8^+^	185	570–2230
CD16/CD56^+^	1458	309–1135
CD19^+^	710	300–774
CD4/CD8^+^	1.8	1–2.2

**Table 2:** Immunoglobulin levels. A reduction of IgG, IgA and IgG_2_ subset was found at diagnosis.

Immunoglobulin levels	Absolute number (mg/dL-UI/mL)	Reference values (5th–95th centiles)
IgG	88	264–1509
IgA	<4	17–178
IgM	187	48–337
IgE	<0.1	≤20
IgG_2_	13	30–170
